# From public healthcare centers to community pharmacies: the new Portuguese seasonal vaccination strategy

**DOI:** 10.3389/fpubh.2026.1747656

**Published:** 2026-05-29

**Authors:** Francisco Goiana-da-Silva, André Peralta-Santos, Miguel Cabral, Duarte Tude-Graça, Pedro Tamudjim-Fonseca, Lara Pinheiro-Guedes, Inês Morais-Vilaça, Diana Costa, Susana Cardoso, João Dionísio, Magda Ornelas, Rafael Vasconcelos, Juliana Sá, Raisa Guedes, Soraia Costa, Filipa Malcata, Rita Moreira, Filomena Cardoso, Jaime Alves, Joel Azevedo, Isabel Cortez, Ema Paulino, Helder Mota-Filipe, Rui Santos Ivo, Rita Sá Machado, João Breda, Hutan Ashrafian, Manuel Pizarro, Ara Darzi

**Affiliations:** 1 Centre for Health Policy, Institute of Global Health Innovation, Imperial College London, London, United Kingdom; 2 Nova Medical School, Universidade Nova de Lisboa, Lisbon, Portugal; 3 Faculdade de Ciências da Saúde, Universidade da Beira Interior, Covilhã, Portugal; 4 Direção-Geral da Saúde, Lisbon, Portugal; 5 NOVA National School of Public Health, Public Health Research Centre, Universidade NOVA de Lisboa, Lisbon, Portugal; 6 Comprehensive Health Research Centre, Universidade NOVA de Lisboa, Lisbon, Portugal; 7 ULS São João, Porto, Portugal; 8 Faculdade de Medicina, Universidade de Lisboa, Lisbon, Portugal; 9 Centre for Public Administration and Public Policies, Institute of Social and Political Sciences, University of Lisbon, Lisbon, Portugal; 10 Escola Nacional de Saúde Pública, Universidade NOVA de Lisboa, Lisbon, Portugal; 11 ULS Tâmega e Sousa, Penafiel, Portugal; 12 ULS de Braga, Braga, Portugal; 13 Ordem dos Farmacêuticos, Lisbon, Portugal; 14 ULS Região de Leiria, Leiria, Portugal; 15 Departamento de Ciências Médicas, Universidade de Aveiro, Aveiro, Portugal; 16 Médis Serviços Saúde, Lisbon, Portugal; 17 Universidade Fernando Pessoa, Porto, Portugal; 18 Ministry of Finance, Lisbon, Portugal; 19 Serviço de Utilização Comum dos Hospitais, Lisbon, Portugal; 20 Associação de Farmácias de Portugal, Lisbon, Portugal; 21 Associação Nacional das Farmácias, Lisbon, Portugal; 22 Faculdade de Farmácia, Universidade de Lisboa, Lisbon, Portugal; 23 Autoridade Nacional do Medicamento e Produtos de Saúde, I. P, Lisbon, Portugal; 24 WHO Athens Quality of Care and Patient Safety Office, World Health Organization Regional Office for Europe, Athens, Greece; 25 Department of Surgery and Cancer, Faculty of Medicine, Imperial College London, London, United Kingdom

**Keywords:** community pharmacies, COVID-19, health systems governance, influenza, seasonal vaccination, vaccination campaign, vaccination strategy

## Abstract

The COVID-19 pandemic significantly altered how countries organize their vaccination strategies. The ongoing discussion now focuses on how these strategies can be integrated into routine vaccination programs. In this context, we illustrate how Portugal has incorporated influenza and COVID-19 vaccinations into community pharmacies in the aftermath of the pandemic. The Portuguese strategy for the 2023–2024 vaccination campaign was led by the Executive Board of the Portuguese National Health Service (SNS) and the Portuguese Directorate-General of Health, in collaboration with over 12 public and private institutions. This strategy included, for the first time, community pharmacies alongside the primary care clinics of the SNS units as the main vaccination points and aligned with the World Health Organization recommendations toward achieving universal health coverage, as it utilized community pharmacies' broad geographical accessibility. Our experience was a success in several metrics. This analysis also highlighted areas for improvement and provided lessons for future campaigns.

## Introduction

Portugal's National Health Service (*Serviço Nacional de Saúde, SNS*) provides universal health coverage, including free-of-charge seasonal influenza and COVID-19 vaccines for all individuals meeting annually defined eligibility criteria established by the Portuguese Directorate-General of Health (Direção-Geral da Saúde, DGS), based on scientific and epidemiological evidence.

Seasonal vaccination plays a vital role in protecting vulnerable populations and preventing health system collapse during the Autumn and Winter. However, vaccination coverage rates remain different across mainland Portugal, as evidenced by the differential influenza coverage rates provided by the Shared Services of the Ministry of Health (Serviços Partilhados do Ministério da Saúde) for the previous season (September 2022–March 2023): North Region: 72 per 100 residents above 60 years (61,194 vaccines administered), Center Region: 50 per 100 residents above 60 years (318,852 vaccines administered), Lisbon and Tagus Valley: 85 per 100 residents above 60 years (523,939 vaccines administered), Alentejo: 42 per 100 residents above 60 years (81,353 vaccines administered), and Algarve: 50 per 100 residents above 60 years (57,025 vaccines administered). Before the 2023/2024 seasonal campaign, influenza and COVID-19 vaccines were primarily administered in SNS primary care units and temporary vaccination centers. As, accessibility is a key barrier, particularly for groups facing disparities in primary care access the World Health Organization (WHO) recognizes the need for partnerships between the public and the private sectors, within mixed health systems, to achieve Universal Health Coverage, and notes that adequate health system governance, based on a complementarity and integrative approach, is crucial to achieve alignment in terms of common health goals and to establish norms and standards to guide these partnerships (1, 2).

Community pharmacies, traditionally overlooked, are gaining recognition for their role in enhancing accessibility due to their widespread presence and trusted staff with healthcare training (3, 4). The Joint guidelines from WHO and the International Pharmaceutical Federation (FIP) on good pharmacy practices stipulate that one of the key functions of pharmacists is the administration of vaccines. Moreover, pharmacists, due to their close relationship with the population, play a crucial role in informing the public about the importance of vaccination and its impact on the wellbeing of the entire community (5). In fact, several countries have already incorporated community pharmacies as venues for vaccine administration (6–11).

As of 2024, pharmacist-led vaccination is authorized in at least 56 countries, with 26 of these also permitting pharmacists to prescribe certain vaccines, a notable increase from just seven countries in 2020. Evidence from countries such as the United States, Canada, Ireland, the United Kingdom, Australia, and France demonstrates that leveraging community pharmacies enhances vaccination coverage and equity (12). Portugal joined this group in 2007, following legislative reforms (Ordinances No. 1429/2007 and No. 97/2018) that expanded the scope of community pharmacies to include immunization services. Under this framework, approximately 2.800 licensed private community pharmacies operate in mainland Portugal, where certified pharmacists, authorized by the Portuguese Pharmaceutical Society and required to renew their certification every 5 years, may administer vaccines not included in the National Vaccination Program, provided they meet specific criteria such as having appropriate facilities and emergency response capabilities. Oversight is ensured by the Portuguese National Authority of Medicines and Health Products (*Autoridade Nacional do Medicamento e Produtos de Saúde, INFARMED*).

The COVID-19 pandemic significantly altered how countries organize their vaccination strategies. The ongoing discussion focuses on how these strategies can be integrated into routine vaccination programs (13). In Portugal, where seasonal vaccination was mainly delivered through primary care clinics of the SNS, the 2023–2024 Autumn-Winter Seasonal Vaccination Campaign (against seasonal influenza and COVID-19) marked a turning point by positioning community pharmacies as the major vaccination sites (14–18).

This initiative aimed not only to mitigate inequities in vaccine access but also to relieve operational burdens on primary care centers, including staff shortages, dependence on overtime, and the use of temporary contracts.

This paper aims to reflect on this new vaccination strategy, discussing the reasons behind its adoption and presenting preliminary findings, while acknowledging the inherent limitations of early data and considering what could be enhanced and closely monitored in the future.

### Global experiences with community pharmacy vaccination strategies

In 1999, in the United States, 22 states allowed pharmacists to administer influenza vaccines to adults (9, 19). By 2014, a national survey found that 93% of the states, cities, and territories provided vaccination services through community pharmacies (20). During the COVID-19 pandemic, the Centers for Disease Control and Prevention formed partnerships with various pharmacy networks to enhance vaccine accessibility, and the Public Readiness and Emergency Preparedness Act has further granted pharmacists authority to prescribe, dispense, and administer COVID-19 and influenza immunizations (21). To fulfill these roles, the federal mandate requires pharmacists to complete an accredited comprehensive training program (21). During the influenza seasons of 2017–2018 and 2020–2021, individuals over 65 primarily resorted to healthcare providers, but pharmacies represented almost half of the sites [45·4% (95% CI: 44·2–46·6) in 2020–2021], serving as an important alternative (9, 22).

In 2015, the English National Health Service (NHS) enabled community pharmacies across the country to administer influenza vaccines to eligible individuals for the 2015–2016 season, building on the previous NHS London Region ‘pharmacy initiative' (23, 24). Results from the pilot test in 2013–2014 did not indicate a significant increase in vaccine uptake (25). Several factors were identified as contributing to the lack of greater success in the first year, including incomplete reporting of pharmacy vaccine administrations into both recording systems, ambiguity in how pharmacies determined individuals' eligibility for vaccination, the program being commissioned close to the flu season's start, and the complexity and variability in the governance structures across trusts, making it challenging to assign responsibilities (24, 25). Despite these challenges, there was an increase in influenza vaccination coverage among one of the most vulnerable groups (individuals at risk and pregnant women). Moreover, the initiative has demonstrated for providing patients with greater convenience and choice in accessing vaccinations, allowing for cost savings. In response to the initial setbacks, the Public Health Commissioning Team considered modifications to the first program (24). Following these adjustments, subsequent years witnessed a positive turnaround, with results showing a substantial increase in vaccination coverage. This success has led to the model's continuation to the present day, with the inclusion of COVID-19 vaccinations and adult routine vaccination under this model (10, 26).

Before the COVID-19 pandemic, several other nations — such as Switzerland and Ireland — were already utilizing community pharmacies in their strategies for seasonal vaccinations (27). Other countries have recently adopted this approach or did so during the pandemic, such as France (10, 28–30). In regard to COVID-19 vaccination campaigns, which began in 2020, by the spring of that year, only a select few European jurisdictions had granted authorization for pharmacists to carry out COVID-19 vaccinations (10, 29, 31, 32).

### Portugal's new seasonal vaccination strategy

In Portugal, the legal framework established by Decree-Law No. 307/2007 allows pharmacies to offer various health promotion services to the population, including the administration of medications and vaccines not covered by the National Vaccination Plan (17). This legislation has enabled pharmacies to offer immunization services since 2007, and no additional primary legislation was required to include them in the new national SNS vaccination strategy. Overall, approximately 7,000 pharmacists were certified in vaccine administration by the Portuguese Pharmaceutical Society (Ordem dos Farmacêuticos), with no additional staff recruitment required. Refresher training on pharmacovigilance, data entry, and emergency response was provided through e-learning modules offered by the ANF and the Pharmaceutical Society.

Expecting a potential drop in the seasonal vaccination coverage for seasonal influenza and COVID-19 (from the previous year coverage of 74% to a predicted range of 51%−74%, for seasonal influenza, and from the previous year coverage of 72·7% to a predicted range of 52%−72·7%, for COVID-19) and in line with the WHO recommended target for adults (≥ 65 years) and high-risk groups, Portugal adapted its strategy to counteract the trend of predicted decline, driven by COVID-19 vaccine fatigue and lower risk perception, maintaining coverage levels above 75% (33).

Since the recommendations by the European Centre for Disease Prevention and Control (ECDC) and the WHO aiming at improving vaccination coverage recommended a focus on the structural determinants of vaccination — (i) physical availability, (ii) cost to users, (iii) geographic proximity, and (iv) communication — the institutions responsible for the 2023–2024 Autumn-Winter Seasonal Vaccination Campaign determined: (34, 35).

**Figure 1:**
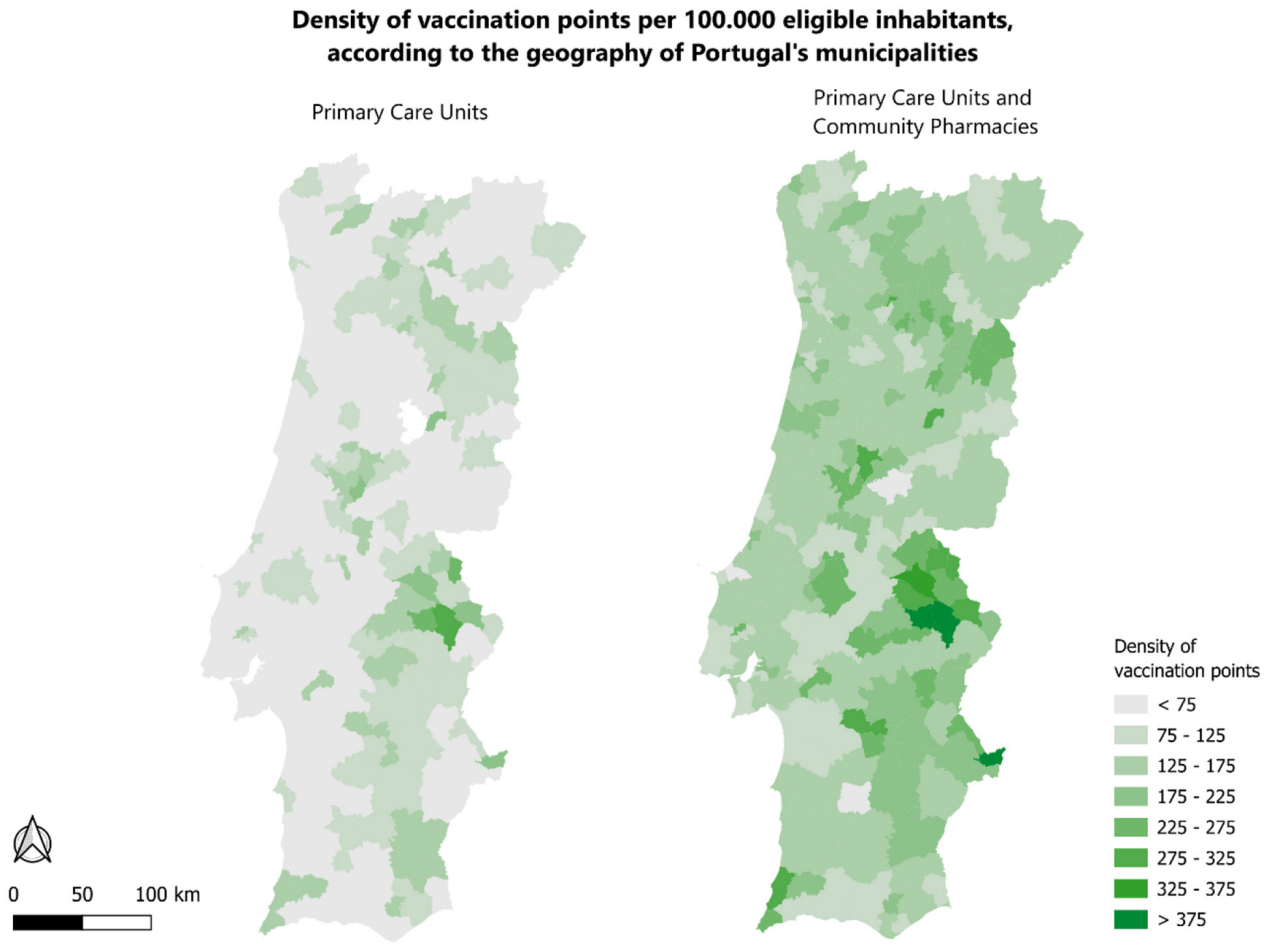
Density of vaccination points per 100,000 eligible inhabitants across Portugal's municipalities. The left map shows primary care units; the right map shows both primary care units and community pharmacies. Darker shades represent areas with a higher density of vaccination points. [The eligible population considered in the figure is people with 60 years or more, eventhough the total eligible population also includes people with 5 or 6 years or more with chronic diseases, for COVID-19 or influenza, respectively; healthcare professionals or other care professionals; others]. (48, 49).

(1) the inclusion of the national network of community pharmacies as main vaccination points in the SNS campaign, improving physical availability and geographic proximity for about 80% of all citizens to be vaccinated free of charge (only those aged 60 or above were eligible for immunization at a community pharmacy) (Figure 1). Pharmacies meeting regulatory and quality criteria such as INFARMED registration, certified staff, suitable facilities, and adherence to the ANF agreement were eligible to participate;
(2) the expansion of the age groups covered by free influenza vaccination to include ages 60–64, increasing accessibility of free vaccination to more than 600,000 Portuguese citizens, in total; (35).
(3) the SNS investment dedicated to the seasonal vaccination communication campaign in the media. This included a nationwide SMS campaign directed to eligible patients were used directed to eligible patients (‘'We have reserved your COVID-19 and flu vaccines. Schedule your appointment at your pharmacy or health center‘'), amplified by over 200 television spots, 400 national, regional, and local radio spots, more than 750 billboards in all mainland districts, and over 3.5 million views on social media pages.

The Portuguese 2023–2024 Autumn-Winter Seasonal Vaccination Campaign was co-led by the Portuguese SNS Executive Board and the Portuguese Directorate General of Health, following a robust and innovative strategy, with strong political backing from the Ministry of Health Governmental team.

This campaign ignited a collaboration involving 12 major public and private entities. For the first time, these collaborated with a unified objective, demonstrating a significant effort of partnership and integration. The involved entities included the SNS Executive Board, the *DGS*, the Portuguese Shared Services of the Ministry of Health (*Serviços Partilhados do Ministério da Saúde, SPMS*), the *INFARMED*, the Common Utilization Service of Hospitals (*Serviço de Utilização Comum dos Hospitais, SUCH*), the Portuguese Central Administration of the Health System (A*dministração Central do Sistema de Saúde*), the National Institute of Health Dr. Ricardo Jorge (*Instituto Nacional de Saúde Doutor Ricardo Jorge*), the Portuguese Pharmacists Society, the Portuguese National Association of Pharmacies (*Associação Nacional das Farmácias*), the Association of Pharmacies of Portugal (*Associação das Farmácias de Portugal*), the Portuguese Association of Pharmaceutical Distributors (*Associação de Distribuidores Farmacêutico*s), and the Portuguese Association of Pharmaceutical Wholesalers (*Associação de Grossistas de Produtos Qu*í*micos e Farmacêuticos*).

Each entity played a critical role in the campaign's success, such as overseeing logistics, regulatory compliance, communication, public health guidance, economic approach, acquisition of vaccines, and integration of health information systems. This integration enabled real-time updates to the national vaccination records and an automatic linkage with INFARMED's National Pharmacovigilance System for adverse events reporting.

Community pharmacies already had direct access to the national vaccination registry (Plataforma VACINAS) through SPMS APIs, allowing them to record vaccine administrations and review patients' influenza and COVID-19 vaccination histories. Access to this system requires digital certification, secure pharmacist authentication, and full compliance with GDPR. Additional responsibilities included laboratorial supervision and professional training.

The planning for this initiative began in January 2023, before the conclusion of the previous seasonal campaign, and was closely monitored by a governance structure, which has been key to its effective execution. This collaboration was of essence to ensure several critical dimensions of the strategy success - (i) health information systems, with secure and real time access to vaccination records and other health information and the ability to register vaccines outside the SNS health units' digital environment through registration in the central vaccination registry (Plataforma *VACINAS)*, which was already in place (ii) safe and effective logistics, with a sophisticated cold chain network to prevent vaccinal waste, (iii) Updates to official training and verification of certification for community pharmacists on vaccine administration and management of complications and the development of best practices, already available since 2007 (iv) system financing changes, from SNS units' global budget to fee-for-service paid directly to community pharmacies, and (v) legislative appropriateness, to include the adopted governance framework.

Vaccine distribution was coordinated by SUCH in close collaboration with pharmaceutical distributors, who ensured capillary nationwide delivery through the existing pharmaceutical supply network. These distributors were responsible for transporting vaccines from central depots to all participating pharmacies under strictly controlled cold chain conditions, thereby guaranteeing timely and equitable access across the country. Distribution planning was informed by demand forecasts generated through the pharmacies' dispensing software, which provided real-time data on appointments and vaccine stock levels. Vaccines were dispatched weekly according to local demand, and stock levels were continuously adjusted to prevent shortages. The SNS maintained overall supply security through centralized procurement and the management of regional buffer stocks.

It is noteworthy that for those vaccinated by the SNS health units, the strategy specifically focused on targeted groups - individuals under 60 with specific pathologies and residents in Long-term Care Facilities for Older Adults (E*struturas Residenciais Para Idosos*), in institutions of the National Network for Integrated Continuous Care (*Rede Nacional de Cuidados Continuados Integrados*) and other institutions such as prisons. Despite that, individuals aged 60 and above could be vaccinated at SNS units, ensuring no opportunity for vaccination was missed if they visited primary healthcare clinics for other reasons or if they wished to be vaccinated there. This approach prioritized the user's autonomy and placed them at the center of care.

### Portugal's findings

Our findings suggest that the implemented strategy improved access to vaccines and convenience for individuals. Vaccination was readily available at participating pharmacies, offering a more comfortable and convenient experience without the need for unnecessary travel. Throughout the campaign, the number of community pharmacies included in the vaccination campaign represented around 88% of all the Portuguese community pharmacies, (36) increasing the number of vaccination points from around 700 in the 2022–23 campaign to more than 3,500 (around a five fold increase) including approximately 2,500 pharmacies and 1,000 SNS primary healthcare units in 2023–24 (35, 37).

It is important to highlight that the peak administration for the seasonal vaccines was attained between the 2nd and the 3rd weeks of October (3 weeks after the beginning of this seasonal vaccination campaign), while, in the previous vaccination season, it occurred only at the 6th week of vaccination. (38, 39). In the 4th week of November, the SNS units started to proactively contact eligible people who had not yet received one of the vaccines, as in previous campaigns, ensuring an efficient, collaborative and active recruitment.

The achievements of the current strategy are particularly evident for influenza vaccination uptake, since there was a decrease in the coverage against COVID-19, despite the same strategy of vaccination being applied to both influenza and COVID-19 vaccines. The decrease in the COVID-19 coverage was also observed in the eligible group below 60 years with chronic conditions, to which the new vaccination strategy in community pharmacies did not apply, in line with other European countries. Considering the expansion of eligibility for free vaccination to the 60–64 age group, this season's influenza vaccination coverage for the entire population with 60 or more years exceeded that from the previous season. According to the DGS, at the final of the 2023–2024 vaccination season, mainland Portugal had an influenza vaccination coverage of 66·3% for this population (compared to 62·4% in the previous season) (40).

When comparing DGS's latest available data, the coverage for the different age groups concerning influenza vaccination seems to be overlapping from this to the last vaccination season (excluding the group from 60 to 64 years old, which was only added in this season). The greatest difference observed between the two vaccination seasons was 0.9 percentage points, for each of all the age groups vaccinated. This is a positive sign in lieu of the expected drop in vaccination coverage for this season.

**Figure 2:**
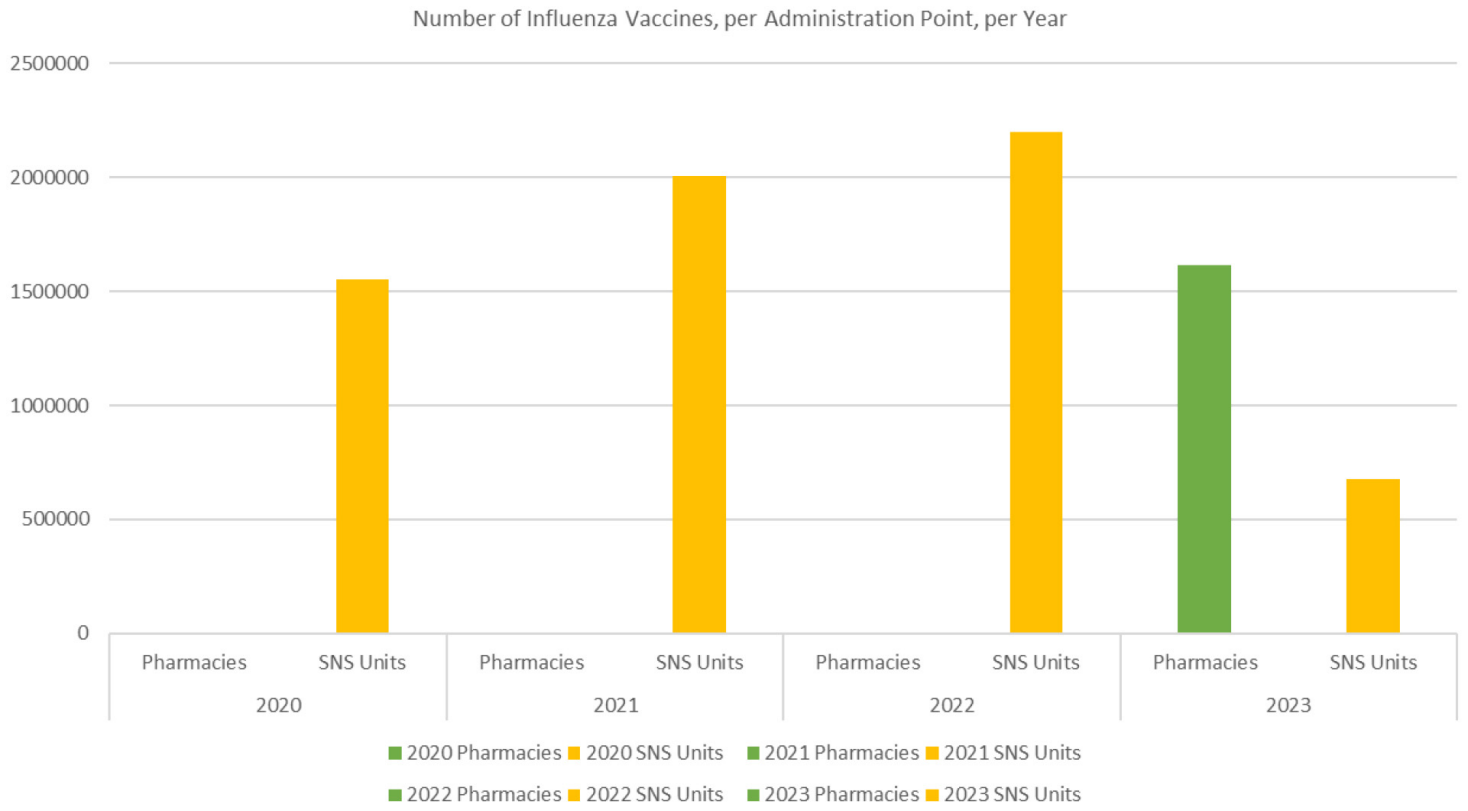
Number of influenza vaccine doses administered at each vaccination point per year. Yellow bars represent the number of flu vaccines administered. Data source: BI-CSP.

It is also relevant to point out the weekly vaccination rate, as the speed of vaccination is paramount to protect the vulnerable population. For several consecutive weeks, the weekly vaccination rate set new records for the country (in non-pandemic settings). The maximum number of influenza vaccines administered per week in the 2022–2023 campaign was about 212,000. In the 2023–2024 campaign, a maximum number of 309,000 influenza vaccines per week was reached, corresponding to a 45.8% increase (35, 38). In this campaign, about 70 and 69% of the influenza and COVID-19 vaccines, respectively, were administered in community pharmacies (38) (Figure 2).

It is noteworthy that there was an additional challenge due to the late delivery of the majority of the influenza vaccines to Portugal, which occurred only in October, delaying the start of the campaign to the last week of September. Comparing the current Portuguese situation to its European counterparts, vaccination coverage against COVID-19 and influenza in Portugal is higher than in countries to which Portugal usually compares to such as Spain and France, according to the latest estimates from DGS (30). In the 2023–2024 season, the Portuguese influenza coverage for 60 years of age or older, as of December 31st, was already 62.4%, even in the face of an international predicted decrease in seasonal vaccination coverage (41). It is noteworthy that the United Kingdom (UK), being the first European adopter of this community pharmacy vaccination strategy, was also the country with the highest influenza coverage from the WHO European Region, despite having experienced some setbacks in the first year of implementation of the strategy (25, 30).

Finally, it is important to note that no major adverse events were identified during the campaign. Minor reactions (e.g., injection-site pain, mild fever) were reported in 0.09% of cases in pharmacies and 0.12% in primary care centers.

### Economic assessment

To enable public funding of the vaccination service provided by pharmacies, the Government published a specific Ordinance establishing reimbursement conditions. Pharmacies were compensated per vaccine dose administered at €2·5 during the 2023–2024 campaign, later increased to €3.00 for 2024–2025. This fee was meant to cover the professional service, including vaccine administration, registration in the central vaccination registry software, materials, waste management, and distribution costs (42).

The remuneration model was negotiated between the Executive Board of the SNS, INFARMED, the DGS, and the ANF. Negotiations considered workload, equipment, consumables, and the operational costs of vaccine delivery in pharmacies. They also incorporated findings from an international evidence review and a benchmarking exercise by ANF comparing remuneration models for pharmacy-based vaccination services in other countries with integrated healthcare systems. Pharmaceutical distributors were also compensated through this fee, ensuring nationwide distribution of vaccines under appropriate cold chain conditions. Funding originated entirely from the SNS central budget, guaranteeing direct public financing and eliminating out-of-pocket costs for the population.

As of 28th of April 2024, there were 1,375,967 COVID-19 vaccines and 1,740,961 influenza vaccines administered in community pharmacies (40).

This campaign changed the way SNS units were organized for the seasonal vaccination process. In the last campaign, centralized services were set up in large facilities (often provided by other stakeholders, such as municipalities), staffed with SNS units' professionals, sometimes on overtime pay or in other conditions that would limit their activities in their allocated SNS units. This year's campaign also allowed savings on overtime work and temporary contracts. In 2022, these measures amounted to about 26 million euros (about 17 million euros for overtime work and 9 million euros for COVID-19 temporary contracts dedicated to the vaccination process). During the entire year of 2022, about 8 million COVID-19 vaccines and, in the last months of the year, 2,339,118 influenza vaccines were administered.

Although opportunity costs were not considered in these estimates, it is important to acknowledge that both health-related costs and opportunities exist. Regarding the costs, it is notable that for the patients, the vaccination moment can be an important interaction between the family nurse and the patient, often used to address other health concerns. Nonetheless, regarding opportunities, this shift potentially frees up more time for family nurses, allowing them to dedicate the consultation time they have with their patients to other individual health promotion programs. This adjustment reflects a balance between optimizing healthcare resource allocation and maintaining quality patient care.

Considering patients' direct costs, it is important to stress that because pharmacies are greater in number and proximity than the usual SNS units, the widespread availability of vaccination options across the country allowed people to choose the closest vaccination point to their homes, jobs, or personal convenience, avoiding personal dislocations during working hours that would imply additional out-of-pocket costs. A study conducted by the Center for Health Studies estimated direct savings for users to be around €2.4 million, which include travel costs, including public transport, cars, and taxis (43). The methodology used was based on the average distance in kilometers from the resident population in mainland Portugal, by municipality, to the nearest official vaccination point (44).

### Equity and social responsibility

It is also important to highlight that certain parts of Portugal have a considerable lack of healthcare professionals. Some areas of the country have more than a third of the population without an assigned primary care team. As such, the possibility of using community pharmacies allows for greater access and reduces pressure on SNS units.

Regarding environmental sustainability, the same study, which estimated fewer patient dislocations, also projected a reduction in CO_2_ emissions by 41%, from 1,252 tons in 2022–23 to 739 tons in 2023–24 (43, 44).

### User's satisfaction

A telephone survey conducted among the Portuguese population assessed perception and satisfaction with the seasonal vaccination campaign, with initial polling before this vaccination season, in September 2023, and a follow-up between January 25 and February 7, 2024. A total of 1,400 valid responses were obtained to the first survey and 1,200 to the second survey (margin of error of 3% and confidence interval of 95%), from a representative panel of the Portuguese population residing in mainland Portugal. The large majority (94.8%) agreed with the expansion to pharmacies. Accessibility and speed were cited as the primary reasons for choosing pharmacies as vaccination sites by 67.4% of the patients, for influenza vaccines, and 63.3%, for COVID-19 vaccines. Furthermore, 88.3% of those vaccinated against influenza at alternative locations expressed willingness to be vaccinated at pharmacies, an opinion shared by 85.0% of those vaccinated against COVID-19 outside pharmacies (37).

When considering all vaccination points, satisfaction scores for this year's seasonal vaccination campaign were high, averaging 4.77 for influenza and 4.76 for COVID-19, on a 1–5 scale, marking an increase from the previous 2022–2023 season, which averages of 4.48 and 4.39, respectively. The current season saw a rise in the proportion of respondents very satisfied with their influenza vaccination (77.9% compared to last year's 50.7%) and COVID-19 vaccination (77.6% up from 47.6%). The study also highlights high satisfaction levels across various dimensions for both health centers and participating community pharmacies: available scheduling (78.3% for influenza, 77.4% for COVID-19), waiting time (78.4% for influenza, 75.6% for COVID-19), healthcare professional competence (78.8% for influenza, 76.9% for COVID-19), and geographical location (77·5% for influenza, 76·4% for COVID-19). While a more detailed satisfaction analysis among those exclusively vaccinated at pharmacies could provide deeper insights, the overall satisfaction improvement could be attributed to the inclusion of pharmacies in the vaccination campaign (37).

### Policy implications and recommendations

The positive findings of the Portuguese 2023–2024 seasonal vaccination strategy, adopted collaboratively by 12 public and private institutions, under the leadership of the SNS Executive Board and the Portuguese Directorate-General of Health, supports the adopted expansion of seasonal vaccination to community pharmacies, which is already being implemented by other countries as a successful framework to maintain high vaccination rates, safety, satisfaction, and increase accessibility for all the eligible citizens.

The expansion of age groups covered by free influenza vaccination to include ages 60–64 demonstrates a proactive approach to address out of pocket expenses and increase equity and vaccination access.

The higher investment in this seasonal vaccination campaign, including widespread television (over 200 spots), radio (over 400 spots), billboards (more than 750), short text messages, and social media advertisements (with over 3·5 million views), highlights the importance of robust communication strategies to promote vaccination. Such campaigns can play a crucial role in raising awareness, dispelling misinformation, and encouraging vaccine uptake among the population. Future initiatives should also be informed by further research on motivation for taking seasonal vaccines already ongoing in some settings (45).

These findings suggest that a multifaceted collaborative approach, in line with recommendations from the ECDC, combining expanded vaccination points, broader age coverage of free vaccination, and comprehensive communication campaigns, can be effective in achieving and maintaining high vaccination coverage (34). Policymakers in other countries may consider adopting similar strategies to enhance their seasonal vaccination programs and mitigate the impact of influenza outbreaks.

It is also important to note that a critical and often-discussed dimension of the 2023–2024 Seasonal Vaccination Campaign was the safety of administering vaccines, particularly concerning potential side effects and adverse reactions of COVID-19 vaccines, in community pharmacies. Despite initial concerns, this risk has been effectively controlled as the vaccines used have been proven safe, pharmacists have received training to manage vaccination processes and handle any adverse reactions, and norms and guidance requiring specific medications and equipment to be available at vaccination points to address potential emergencies have been proposed and implemented. Furthermore, a robust surveillance system, managed by INFARMED, ensures continuous monitoring and immediate response to any adverse events, further guaranteeing the safety and effectiveness of this vaccination campaign.

Continued monitoring and evaluation of the impact of these policy changes are crucial to assessing their long-term effectiveness and identifying areas for further improvement. Regular assessment of vaccination coverage rates and public perceptions can provide valuable insights into the success of these interventions and inform future policy decisions in public health and seasonal vaccination campaigns.

### Limitations and lessons learned

The strategy for the 2023–2024 seasonal vaccination campaign, which required significant planning and dedication, was essential to align Portugal with international collaborative vaccination strategies. However, we recognize some limitations that might impact our findings. The analyzed data of the various countries were not disaggregated, as it consisted mainly of publicly available figures that lack detailed breakdowns by individual characteristics to discern where vaccination gaps were most relevant. Additionally, the lack of an uniform vaccination model and the lack of free vaccine distribution policies across different countries pose a challenge to making comparisons between vaccination strategies globally.

Vaccination hesitancy is a current and future challenge. How professionals involved in the vaccination can decrease hesitancy is crucial to maintain high vaccination rates, especially in hard-to-reach populations (46).

Overall, the current strategy allowed a smaller allocation of financial resources, while allowing better conditions for SNS's professionals and services, as well as patient satisfaction. Therefore, this strategy contributes to a greater value-based healthcare organization, in line with the current framework of the European Commission (47).

Regarding the implemented strategic adjustments, some operational factors may have played a role in achieving outcomes. The identified challenges are similar to the constraints faced by the UK in its first year of this strategy, thus providing an indication that further benefits will continue to occur with the maintenance and development of the strategy (25). Nonetheless, we should note that this was the first year of implementation of a collaborative, proximity strategy of vaccination that was never seen in Portugal.

In the coming years, it would be important to ensure early guidance on the operational details of the seasonal vaccination campaigns, guaranteeing that both the SNS units and community pharmacies have time for local planning and implementation. An example could be an earlier or staggered start (older age groups first) for the proactive recruitment of the eligible people who have not yet been vaccinated. Additionally, it is vital to ensure that pharmacists continue to receive training for administering injectables, the health information systems are maintained, validated or even evolve with new features, and the logistics of vaccine distribution are assured. Investment in a strong, planned communication strategy to increase populations's health literacy is also crucial to enhance adherence and maintain high vaccination coverage.

## Conclusions

The innovative adaptations to the Portuguese seasonal vaccination strategy, implemented under the leadership of the SNS Executive Board, in a partnership with the Portuguese Directorate-General of Health, for the 2023–2024 season, have demonstrated promising results. The political impact on various sectors was profound, focusing on the interests of the users and the State. This approach not only enhanced the efficiency and reach of the vaccination campaign but also exemplified a model of integrated public health management. Comparing the current Portuguese situation to its European counterparts, vaccination coverage against COVID-19 and influenza in Portugal is currently higher than that in countries such as Spain and France. These findings underscore the importance of continuous monitoring and evaluation of vaccination strategies until the end of the vaccination campaign, as well as the potential benefits of adopting similar approaches in other countries where they are not yet implemented.

Community pharmacies have shown to be safe vaccination points with trained professionals, offering convenient and easy access for users due to their proximity and geographical availability. Moreover, this new strategy showed favorable economic impacts, allowed for the use of the already established health information systems, and ensured a safe and effective distribution network. Given these benefits, the future challenge is to expand the mission of the community pharmacies, serving as vaccination points for SNS-covered vaccines (free of charge), under the Portuguese National Vaccination Plan, in addition to seasonal vaccines, particularly for adult immunizations such as tetanus and diphtheria (administered at ages 25, 45, 65, and subsequently every 10 years), consequently reducing the SNS workload. Further research will be essential to assess the long-term impacts of this new strategy and inform future policy decisions in public health and vaccination programs.

## Data Availability

The original contributions presented in the study are included in the article/supplementary material, further inquiries can be directed to the corresponding author.
